# Enhancement of Electrical Properties of Sol–Gel Indium–Tin–Oxide Films by Microwave Irradiation and Plasma Treatment

**DOI:** 10.3390/mi12101167

**Published:** 2021-09-28

**Authors:** Sung-Hun Kim, Won-Ju Cho

**Affiliations:** Department of Electronic Materials Engineering, Kwangwoon University, 20 Kwangwoon-ro, Nowon-gu, Seoul 01897, Korea; tjdgns0721@naver.com

**Keywords:** sol–gel indium–tin–oxide film, microwave irradiation, plasma treatment, ion bombardment

## Abstract

We proposed the enhancement of the electrical properties of solution-processed indium–tin–oxide (ITO) thin films through microwave irradiation (MWI) and argon (Ar) gas plasma treatment. A cost- and time-effective heat treatment through MWI was applied as a post-deposition annealing (PDA) process to spin-coated ITO thin films. Subsequently, the sheet resistance of MWI ITO thin films was evaluated before and after plasma treatment. The change in the sheet resistance demonstrated that MWI PDA and Ar plasma treatment significantly improved the electrical properties of the ITO thin films. Furthermore, X-ray photoelectron spectroscopy and X-ray diffraction analyses showed that the electrical properties of the ITO thin films were enhanced by the increase in oxygen vacancies due to the ion bombardment effect of high-energy plasma ions during Ar plasma treatment. Changes in the band gap structure of the ITO thin film due to the ion bombardment effect were also analyzed. The combination of MWI PDA and Ar plasma treatment presents new possibilities for improving the high-conductivity sol–gel ITO electrode.

## 1. Introduction

Indium tin oxide (ITO) is one of the key materials for transparent conductive oxides in optoelectronics. ITO provides major advantages, i.e., high optical transmittance in the visible wavelength region, stable chemical properties, easy patterning ability, and excellent substrate adhesion. Hence, it is widely used as a transparent electrode in electro-optical devices such as organic light emitting diodes, solar cells, and image sensors [1,2,3,4]. Various methods of fabricating ITO thin films are being studied, such as magnetron sputtering, spray pyrolysis, electron beam deposition, pulse laser deposition, chemical vapor deposition, and solution process deposition [5,6,7,8,9,10,11]. Among these, solution-process-based ITO thin films have drawn attention because of their simple and low-cost fabrication process without expensive high-vacuum equipment, easy ratio adjustment, and homogeneity [12]. Various processes are being investigated to improve the conductivity of sol–gel ITO thin films to ensure their practicality, e.g., post-deposition annealing (PDA), passivation, plasma surface treatment, excimer laser crystallization, and ultraviolet–ozone photo treatment [13,14,15,16,17]. Plasma surface treatment uses high-energy electrons to change the chemical bonding on the surface of a material. In particular, as the plasma-based process is simple, ecofriendly, and low temperature, it has been applied for the precision cleaning of semiconductor surfaces and photoresist ashing processes [18]. Numerous gases are used for plasma surface treatment depending on its purpose [19,20,21,22]. Argon (Ar) causes high-energy electron collision on a surface during plasma treatment, thereby increasing oxygen vacancies. This phenomenon, referred to as the ion bombardment effect, has been observed in several amorphous oxide semiconductors [20,23]. In general, the conductivity of ITO films can be described by the increase in the carrier concentration with the number of oxygen vacancies. Low-resistance ITO thin films can be obtained by increasing the carrier concentration [24]. There have been reports of studies on improving conductivity by applying Ar plasma treatment to ITO thin films, but most of them are conducted on ITO thin films processed with conventional furnace annealing (CFA) for PDA [25,26]. However, in our previous study we applied microwave irradiation (MWI) to sol–gel ITO thin films, a high-efficiency heat treatment with a shorter time than the CFA heat treatment [27]. MWI heat treatment affects the molecular level of a material through the 2.45 GHz electromagnetic wave. The interaction of molecular dipoles with microwaves results in rotation of the dipoles, and the energy is converted into heat for rotation by internal resistance. Therefore, MWI is an efficient heat treatment method that directly transfers heat to materials through electromagnetic waves, and since metal salts efficiently absorb 2.45 GHz microwaves, it is widely studied as a promising technology for oxide semiconductors [28,29,30,31].

In this study, we prepared low-cost sol–gel ITO thin films and improved their electrical properties through MWI PDA and Ar plasma treatment. Additionally, to identify the effects of MWI PDA and Ar plasma treatment, CFA and O_2_-plasma-treated ITO thin film samples were prepared. The sheet resistance of the PDA and plasma-treated ITO thin films was measured to evaluate their electrical properties. In addition, X-ray photoelectron spectroscopy (XPS), X-ray diffraction (XRD), and optical transmittance measurements were carried out to analyze the chemical state, crystalline state, and band gap model of the sol–gel ITO thin films, respectively. The improvement in the electrical properties of the ITO thin films was attributed to the removal of residual organic contaminants by MWI and the increase in oxygen vacancies due to the ion bombardment effect of high-energy plasma ions during Ar plasma treatment. The results of this study demonstrate that high-conductivity transparent ITO electrodes can be developed by performing MWI PDA and Ar plasma treatment on solution-processed ITO thin films, thereby enabling high-performance optoelectronics.

## 2. Materials and Methods

**Figure 1:**
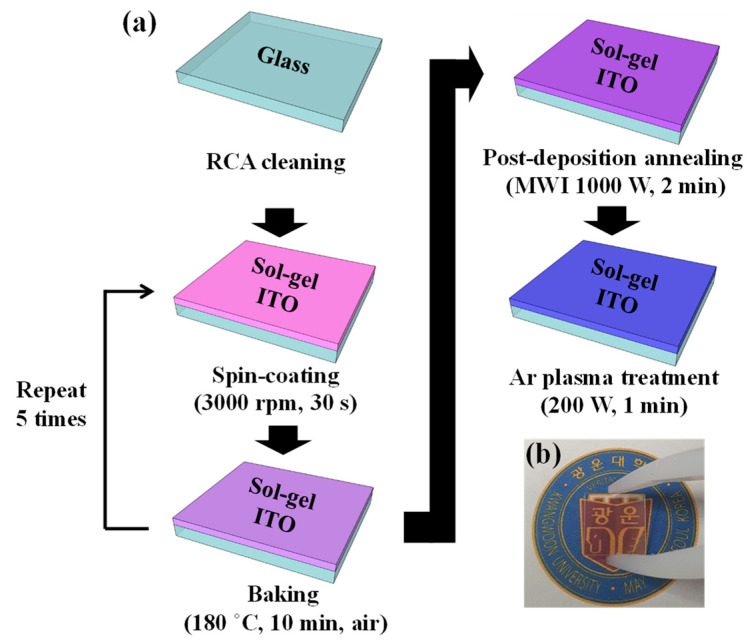
(**a**) Fabrication steps for sol–gel indium–tin–oxide (ITO) thin films; (**b**) photograph of the ITO-coated glass sample (MWI: microwave irradiation).

An ITO precursor solution was prepared via a sol–gel reaction. Anhydrous indium trichloride (InCl_3_, purity = 99.9%; Sigma Aldrich, Saint Louis, USA) and anhydrous tin chloride (SnCl_4_; Sigma Aldrich) were dissolved in 20 mL of 2-methoxyethanol (C_3_H_8_O_2_; Sigma Aldrich). To improve the stability of the coating, 2.5 mL of monoethanolamine (C_2_H_7_NO; Sigma Aldrich) was added at room temperature. The mixture was then stirred using an electronic agitator at 50 °C for 2 h in a closed vessel. The prepared ITO precursor solution was spin coated at 3000 rpm for 30 s on Corning 7059 glass substrates (Corning Inc., New York, USA), which were cleaned using the Radio Corporation of America process. The solvent was removed by baking the substrates in an oven at 180 °C in air for 10 min. The coating process was repeated five times to create five layers. The thicknesses of the layers were measured as ~100 nm using the DektakXT Bruker stylus profiler (Bruker, Hamburg, Germany). The fabrication steps for the ITO samples are summarized in Figure 1a, and the photograph of the ITO-coated glass thin film is shown in Figure 1b.

**Figure 2:**
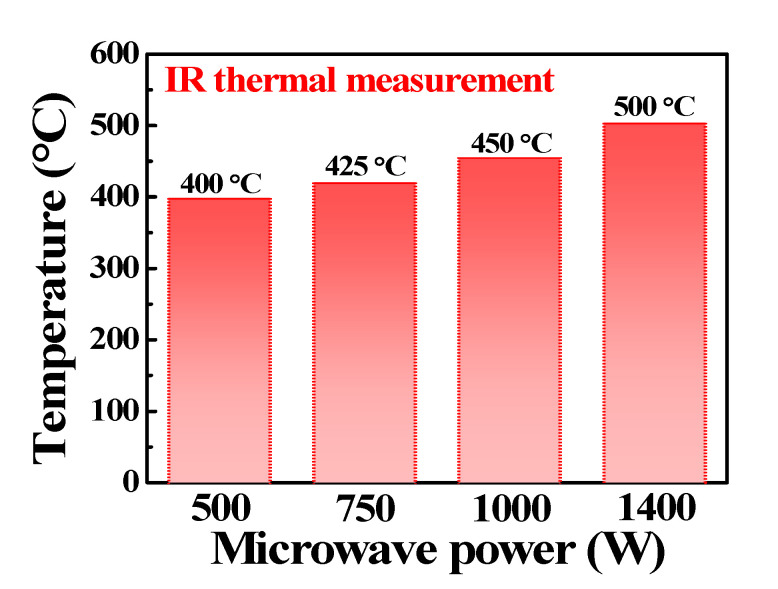
Variation in the temperature of ITO-coated glass substrate with microwave power.

A cost-effective and low-thermal budget MWI technique was applied for PDA. The MWI process conditions were as follows: a rated power of 1000 W was delivered for 2 min at a microwave frequency of 2.45 GHz in an O_2_ atmosphere. Figure 2 shows the average temperature of the ITO thin film vs. the microwave power. The temperature was measured using an infrared thermometer. The temperature increased almost linearly with the microwave power. For comparison, CFA was applied to ITO thin films in an O_2_ atmosphere at 450 °C for 30 min. Under the same temperature condition, the CFA method transfers the heat slowly by radiation, convection, and conduction mechanisms from an external heating source, but MWI has a shorter heat treatment time than CFA in a way that the material absorbs electromagnetic energy volumetrically and converts it into heat by coupling with microwaves [32].

After PDA, Ar plasma treatment was performed under various conditions to find the optimized conditions for improving the electrical properties of the ITO film. Furthermore, O_2_ plasma treatment was performed to compare the properties of the films. The plasma treatment processes were conducted by employing reactive ion etching (RIE) equipment under the following conditions: 50 sccm of Ar and O_2_ were individually operated at a vacuum of 300 mTorr in a range of 1–4 min at RIE power of 50, 100, 150, and 200 W. The electrical properties of the ITO films were investigated by measuring the sheet resistance (*R_s_*) using a four-point probe (Advanced Instrument Technology Inc, Cumming, GA, USA). The optical properties of the films were investigated by measuring the transmittance using an ultraviolet–visible spectrophotometer (Agilent Technologies, Santa Clara, CA, USA) in a wavelength range of 300–1000 nm. The binding energy and the crystallization of the films were analyzed via XPS and XRD, respectively.

## 3. Results and Discussion

**Figure 3:**
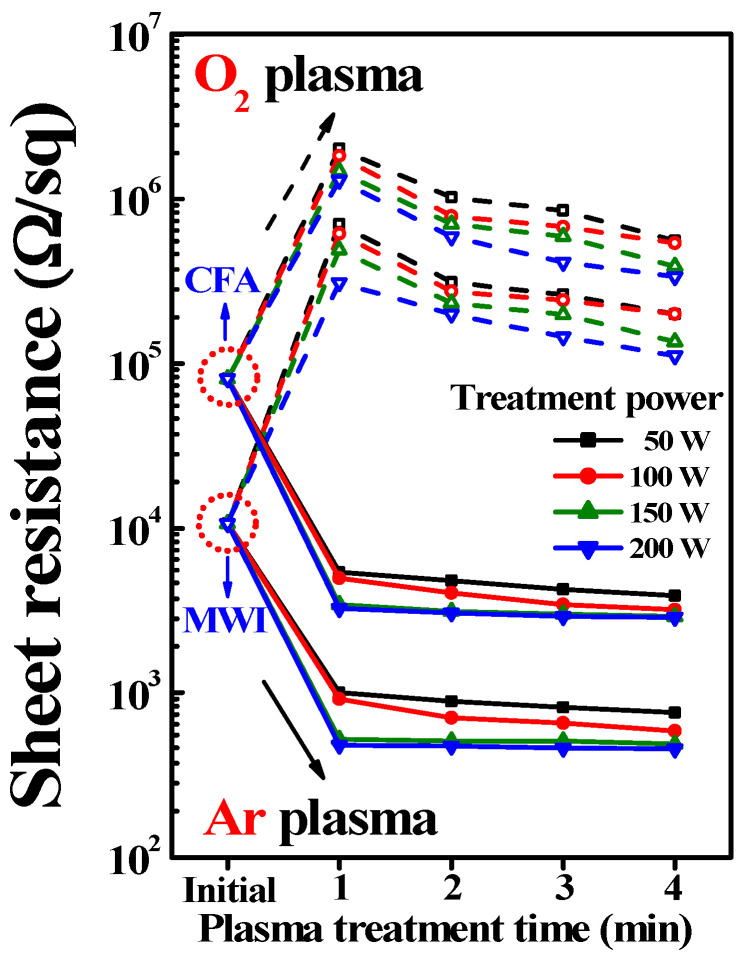
Variation in sheet resistance (log scale) with plasma treatment time and power for MWI and conventional furnace annealing (CFA) ITO films.

Figure 3 shows the plot of *R_s_* vs. the plasma treatment time for the ITO films to which MWI and CFA (450 °C) were applied, respectively. The microwave power was 1000 W. The initial *R_s_* of the MWI and CFA ITO films before plasma treatment was 1.06 × 10^4^ Ω·sq^−1^ and 8.09 × 10^4^ Ω·sq^−1^, respectively. Even in the initial state before plasma treatment, the conductivity of the ITO film was about 8 times higher in MWI than CFA. This is because, as previously reported in our study [27], MWI is superior to CFA in terms of precursor and solvent decomposition owing to the high transfer efficiency of microwave energy. Furthermore, MWI is more effective in removing residual organic contaminants and obtaining a high level of solution condensation and metal oxide film densification [32,33,34,35]. After PDA, *R*_s_ increased with the plasma treatment time and power in the case of O_2_ plasma treatment. However, in the case of Ar plasma treatment, *R*_s_ considerably decreased as the plasma treatment time and power increased. The best *R*_s_ was obtained at a plasma treatment power of 200 W, and it became saturated after 1 min. Therefore, a sufficient increase in conductivity can be ensured when *R_s_* is 4.80 × 10^2^ Ω·sq^−1^ at 200 W after 1 min, and the conductivity is about 6 times higher than under CFA condition. This confirms that conductivity can be significantly improved by employing MWI and Ar plasma treatment.

**Figure 4:**
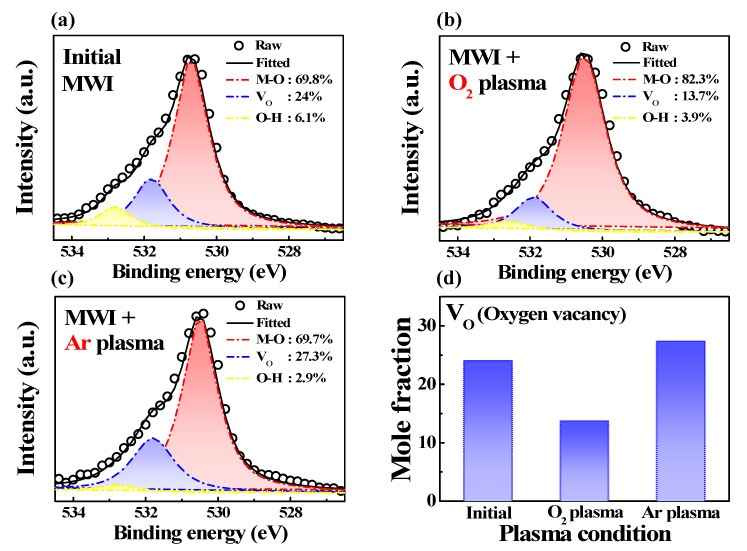
X-ray photoelectron spectroscopy patterns of MWI ITO thin films (**a**) before plasma treatment, (**b**) after O_2_ plasma treatment, and (**c**) after Ar plasma treatment; (**d**) mole fraction of oxygen vacancies.

Various physical analyses were conducted to examine the improvement in the conductivity of the ITO film due to Ar plasma treatment. The change in the composition caused by plasma treatment was measured through XPS analysis. O1s spectra can be used to analyze the binding energy with oxygen, which is an important part of conductivity in ITO films [36]. O1s spectra can be deconvoluted into three components using Gaussian shaped peaks. The component at 530.5 eV corresponds to oxygen in the In_2_O_3_ lattice without oxygen vacancies (M-O). The component at 531.9 eV corresponds to oxygen in the In_2_O_3_ lattice with oxygen vacancies (V_O_) and that at 532.8 eV corresponds to the oxygen of free hydroxyl groups attached to indium ions (O-H) [36,37,38]. Figure 4 shows the XPS spectra of the MWI ITO film after Ar and O_2_ plasma treatments. The XPS spectra of the MWI ITO film before plasma treatment are shown in Figure 4a. The XPS spectra of the film after O_2_ and Ar plasma treatments are shown in Figure 4b,c, respectively. Plasma treatments were carried out at 200 W for 1 min. The 532.8 eV component decreased after plasma treatments. This is known to be mainly caused by surface cleaning during plasma treatment [17]. The 531.9 eV component increased after Ar plasma treatment but decreased after O_2_ plasma treatment. The change in the mole fraction of oxygen vacancies is shown in Figure 4d. During O_2_ plasma treatment, the abundant high-energy O_2_ gas plasma affected the oxygen component of the surface and increased the binding energy, thereby reducing oxygen vacancies [39,40]. On the contrary, during Ar plasma treatment, oxygen vacancies increased because the binding force with oxygen in the In_2_O_3_ lattice was weakened by the ion bombardment effect. This has been observed in various other oxide semiconductors [20,41]. The ion bombardment effect improves conductivity because the carrier concentration increases with oxygen vacancies.

**Figure 5:**
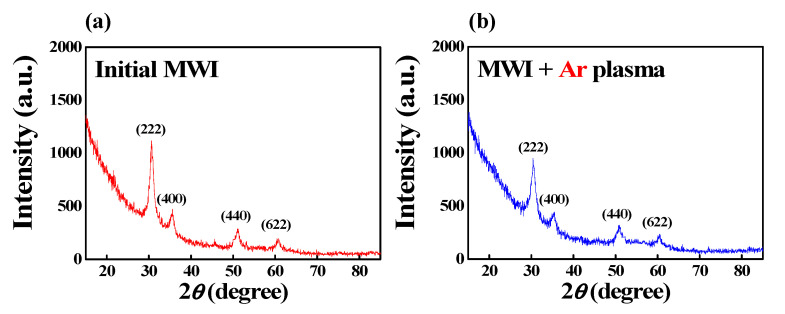
X-ray diffraction results of MWI ITO films (**a**) before and (**b**) after Ar plasma treatment.

However, the reduction in the binding force caused by the ion bombardment effect may affect the crystallinity of the ITO film. Thus, the XRD patterns of the ITO films were analyzed to investigate crystallinity in detail. Figure 5 shows the XRD patterns of the MWI ITO films. The stable peaks at (222), (400), (440), and (622) represent the typical XRD peaks for ITO films. In particular, the preferential growth of the ITO film occurred along the (222) plane, which strongly depends on the preparation conditions [42]. Crystallinity can be quantitatively determined by extracting the grain size at the main (222) peak. The average grain size is obtained through the well-known Scherrer formula given by $Dp=\frac{0.9\lambda }{\beta \;\cos\theta },\;$ where *d* is the average grain size, *β* is line broadening in radians, *θ* is the Bragg angle, and *λ* is the X-ray wavelength [43]. The grain size calculated at the (222) peak of the MWI ITO film without plasma treatment was 9.56 nm. The grain size after Ar plasma treatment reduced to 8.32 nm. Therefore, it is confirmed that the ion bombardment effect during Ar plasma treatment decreases the binding force with oxygen and slightly reduces crystallinity.

**Figure 6:**
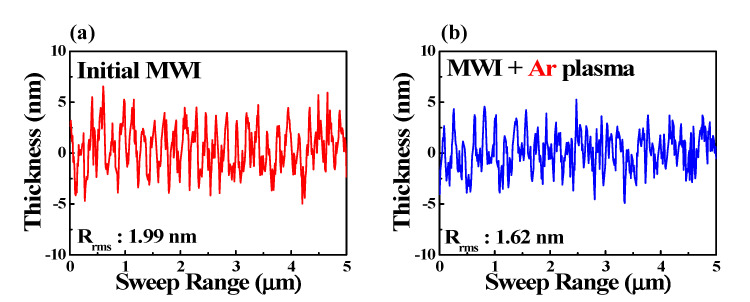
Surface roughness properties of MWI ITO films (**a**) before and (**b**) after Ar plasma treatment.

Figure 6 shows the surface roughness of the MWI ITO thin film in the 5 μm range measured with a stylus surface profiler (Dektak XT, Bruker). The root-mean-square (rms) of the MWI ITO thin film was 1.99 nm in the initial MWI state and 1.62 nm after Ar plasma treatment. The surface roughness was slightly reduced by Ar plasma treatment. This tendency has been reported in previous studies on plasma-treated ITO thin films [44,45]. The improvement in surface roughness is considered to be due to the crystallinity of the ITO thin film, which can be inferred from the XRD data in Figure 5. Therefore, the Ar plasma treatment contributes to the improvement of the conductivity and surface properties of the ITO thin film, which greatly affects the reliability of optoelectronic devices.

**Figure 7:**
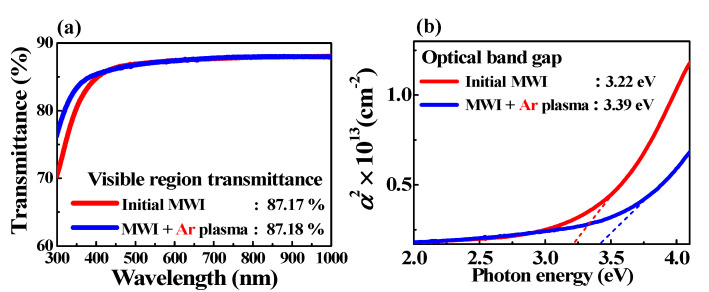
(**a**) Optical transmittance spectra of MWI ITO films; (**b**) optical band gap calculated using optical absorption coefficient.

The ion bombardment effect is also confirmed in the optical characteristics of Ar-plasma-treated ITO films. Figure 7a shows the optical transmittance spectra of the MWI ITO films. The transmittance was obtained by normalizing the transmittance of the initial glass substrate to 100%. Figure 7b shows the plot of the optical absorption coefficient (α) vs. the optical energy of Ar-plasma-treated and untreated ITO films. The average transmittances of the initial MWI ITO films and Ar-plasma-treated ITO films in the visible region (400–800 nm) were 87.17% and 87.18%, respectively. In addition, it is possible to confirm the ion bombardment effect on the basis of the optical band gap difference, which is calculated using α. α is obtained from the optical transmittance spectral data using $\alpha =\frac{1}{d}ln\left(\frac{1}{T}\right)$, where *d* is the film thickness [46]. The optical band gap (*E*_g_) is calculated using ${\left(\alpha hv\right)}^{\frac{1}{n}}=A\left(hv-E_{g}\right)$, where *A* is a constant and *hν* is the incident photon energy. *n* depends on the type of transition, where *n* = 1/2 and 2 are used for direct and indirect transitions, respectively [47]. Given that the ITO film has a direct band gap, *E*_g_ can be extracted from the plot of (*αhν*)^2^ vs. *hν*. The *E*_g_ of the MWI ITO film was obtained as 3.22 eV, and it increased to 3.39 eV after Ar plasma treatment. Transmittance and band gap changes according to MWI, CFA, and Ar plasma treatment are shown in Table 1.

Therefore, Ar plasma treatment slightly improved the optical properties of the ITO films. In addition, the increase in optical band gap by Ar plasma treatment was caused by the ion bombardment effect [48,49]. Ion bombardment weakens the binding force with oxygen in the In_2_O_3_ lattice and thus increases oxygen vacancies. This dominantly leads to a Burstein–Moss effect that widens the optically measured band gap [50,51,52]. This explains why the optical band gap increases after Ar plasma treatment.

**Figure 8:**
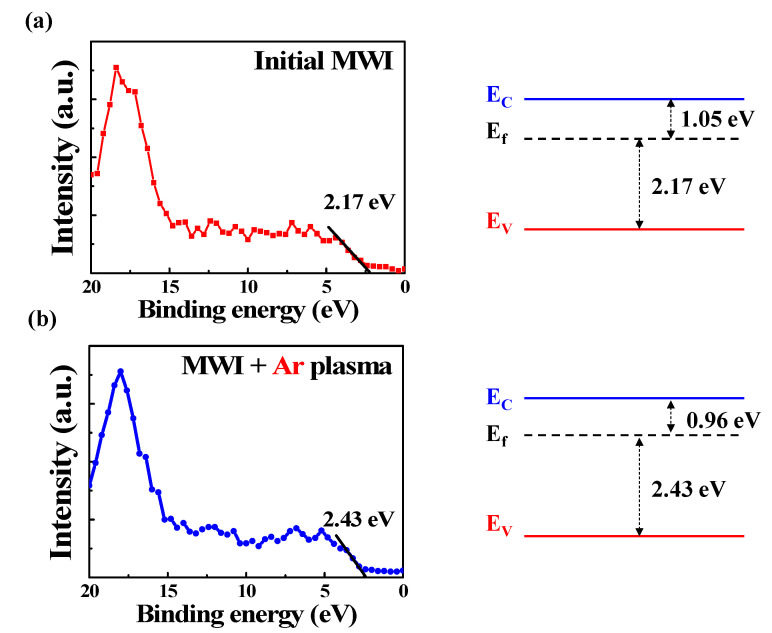
XPS valence band spectra and energy band structure of MWI ITO films (**a**) before and (**b**) after Ar plasma treatment.

Figure 8 shows the XPS spectra near the valance band and the band structure model of the ITO film. To investigate the energy band structure of the ITO film, the energy level difference between the valence band maximum and the Fermi energy level (*E*_F_–*E*_VBM_) was extracted from the XPS valence band spectra [53]. The *E*_F_–*E*_VBM_ values of the initial MWI ITO films and Ar-plasma-treated ITO films were determined to be 2.17 eV and 2.43 eV, respectively. Subsequently, through the *E*_g_ value extracted from optical absorption coefficient in Figure 7, the energy band structures of the ITO films could be estimated using these *E*_F_–*E*_VBM_ and *E*_g_ values. After Ar plasma treatment on the initial MWI ITO film, it can be seen that the *E*_g_ value increased and the *E*_f_ approached the conduction band (*E*_c_). This proves the effects of ion bombardment during the Ar plasma treatment. The *E*_g_ value increase is due to the Burstein–Moss effect, and the *E*_c_ approaches of *E*_f_ are due to the increase in oxygen vacancies (electron concentration), respectively [54].

## 4. Conclusions

We investigated a method of improving the electrical properties of sol–gel ITO films by adapting Ar plasma treatment and MWI. The sheet resistance of MWI and CFA ITO thin films was compared. The sheet resistance of the ITO films to which MWI was applied at 1000 W was considerably less than that of the ITO films to which CFA (450 °C) was applied. Furthermore, Ar plasma treatment significantly decreased the sheet resistance of the ITO films, whereas O_2_ plasma treatment increased the sheet resistance. The cause of this change in the sheet resistance was identified by analyzing binding energy through XPS. XRD analysis confirmed that Ar plasma treatment significantly improved the conductivity of the ITO films and slightly decreased crystallinity owing to the ion bombardment. In addition, The Burstein–Moss effect and oxygen vacancies increase caused by ion bombardment were verified by extracting the band structure through optical and XPS characterization. The results of this study suggest a breakthrough method for using sol–gel ITO thin films as high-efficiency, low-resistance backplane electrodes in flexible see-through displays.

## Figures and Tables

**Table 1:** Optical properties of sol–gel ITO thin films according to treatment condition.

Treatment Condition	Transmittance (400–800 nm)	Optical Band Gap
MWI	87.17%	3.22 eV
MWI + Ar plasma	87.18%	3.39 eV
CFA	87.08%	3.31 eV
CFA + Ar plasma	87.11%	3.54 eV

## Data Availability

Not applicable.
